# Potential of Cyanine Derived Dyes in Photodynamic Therapy

**DOI:** 10.3390/pharmaceutics13060818

**Published:** 2021-05-31

**Authors:** Natalia Lange, Wojciech Szlasa, Jolanta Saczko, Agnieszka Chwiłkowska

**Affiliations:** 1 Faculty of Medicine, Wroclaw Medical University, Mikulicza-Radeckiego 5, 50-345 Wroclaw, Poland; natalia.lange@student.umed.wroc.pl (N.L.); wojciech.szlasa@outlook.com (W.S.); 2 Department of Molecular and Cellular Biology, Faculty of Pharmacy, Wroclaw Medical University, Borowska 211A, 50-556 Wroclaw, Poland; jolanta.saczko@umed.wroc.pl

**Keywords:** cyanine dyes, photodynamic therapy, cancer therapy, irradiation

## Abstract

Photodynamic therapy (PDT) is a method of cancer treatment that leads to the disintegration of cancer cells and has developed significantly in recent years. The clinically used photosensitizers are primarily porphyrin, which absorbs light in the red spectrum and their absorbance maxima are relatively short. This review presents group of compounds and their derivatives that are considered to be potential photosensitizers in PDT. Cyanine dyes are compounds that typically absorb light in the visible to near-infrared-I (NIR-I) spectrum range (750–900 nm). This meta-analysis comprises the current studies on cyanine dye derivatives, such as indocyanine green (so far used solely as a diagnostic agent), heptamethine and pentamethine dyes, squaraine dyes, merocyanines and phthalocyanines. The wide array of the cyanine derivatives arises from their structural modifications (e.g., halogenation, incorporation of metal atoms or organic structures, or synthesis of lactosomes, emulsions or conjugation). All the following modifications aim to increase solubility in aqueous media, enhance phototoxicity, and decrease photobleaching. In addition, the changes introduce new features like pH-sensitivity. The cyanine dyes involved in photodynamic reactions could be incorporated into sets of PDT agents.

## 1. Introduction

### 1.1. Basics of Photodynamic Therapy

**Figure 1:**
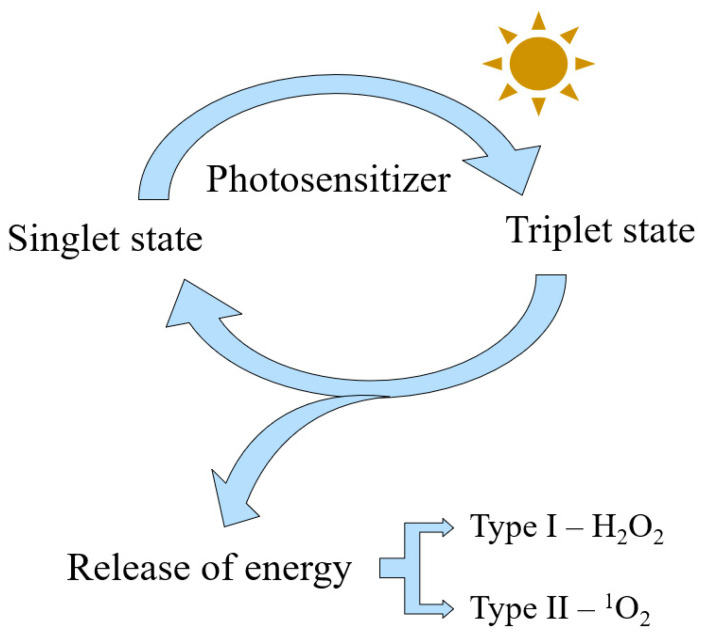
The mechanism of a photosensitizer action in photodynamic therapy.

Photodynamic therapy (PDT) is a low-invasive therapy, that destroys cancer cells through the generation of reactive oxygen species (ROSs). PDT involves a photosensitizer (PS), administered topically or intravenously, a light source and oxygen in the targeted tissue [1]. In the dark, the PS remains in its base energy state. However, the absorption of light at the appropriate wavelength moves the PS to an excited state. An excess of absorbed energy leads to its release and reaction with the oxygen in the tissue, thus inducing the formation of ROS [2]. The photodynamic reaction might propagate in two main ways [3]. The Type I mechanism of photodynamic reaction includes the production of highly reactive intermediates, mainly hydrogen peroxide H_2_O_2_ and •OH. Type II involves the formation of oxygen in the singlet state ^1^O_2_ (Figure 1) [4]. The short half-life of free radicals determines the limited diffusion distance of these high cytotoxic molecules [5]. The dysregulation of the homeostasis free radicals induces irreversible oxidation of proteins, nucleic acids, fatty acids, cholesterol and organelles of the tumor cells. The process leads to loss of function and eventually to the death of the irradiated cells depending on the conditions of the therapy, especially the cellular localization of the photosensitizer [5]. Death might also arise from an induced immune response as well as from the destruction of vessels supplying the tumor with nutrition [6].

### 1.2. Advantages and Disadvantages of PDT

Several advantages of PDT make it a promising treatment against malignancies. Selectivity of the PS towards cancer cells makes it applicable for curative treatment, early-stage tumors, and reducing the size of inoperable tumors. Additionally, as a local low-invasive procedure, PDT has minimal systemic side effects compared with standard chemotherapy or radiotherapy. PDT can also lead to the eradication of the tumor vasculature [5], which will be described in Section 1.5.

Another point in its favor is that PDT may trigger various immune responses to cancerous cells [7]. Primarily, if the irradiated cells undergo necrosis, an acute inflammatory response mediates the removal of the dead tissue [8]. Then, dendritic cells (DCs) absorb the cancer-related antigens through phagocytosis and present it to the immune-system cells, thus triggering the systemic inhibitory response to cancer cells [9].

**Figure 2:**
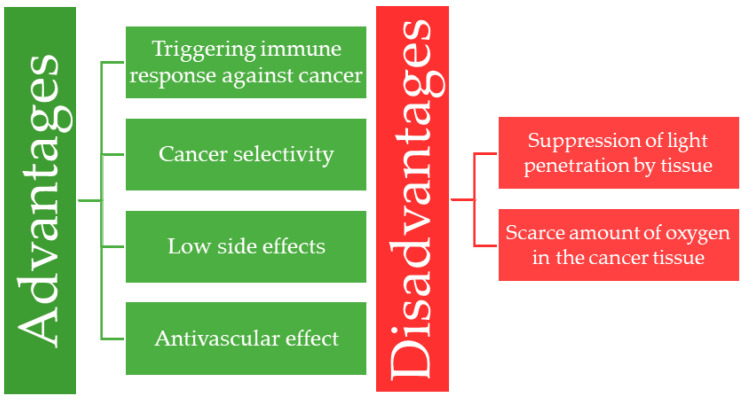
Summary of the main advantages and disadvantages of photodynamic therapy.

The penetration of light at an appropriate wavelength to activate the FDA-approved PSs (Porfimer sodium, Temoporfin, Verteporfin [10]) can be suppressed by the tissue, thus putting deep-seated tumors out of reach. To overcome this problem, PSs, that absorb light from the NIR-I could be applied. Another obstacle is the low oxygenation of the tumor microenvironment. Namely, when cancer cells are in hypoxia [11], PDT may be rendered ineffective due to the lack of substrate for ROS generation. The main advantages and disadvantages of PDT are shown in Figure 2, whereas a broad reference to the efficacy and application of the PDT is reviewed by Agostinis et al. [12].

### 1.3. Desired Characteristics of the Photosensitizer

A PS should be characterized by low dark toxicity and selective accumulation in the tumor cells. The compound should be highly susceptible to irradiation and generate as many ROS as possible in the tumor environment [13]. Also, an ideal PS should localize outside the nucleus to avoid the pro-cancerous mutations in the DNA [1]. Preferably, an ROS should be generated in the mitochondria or lysosomes, but an especially effective targeting combination involves both of them [14]. Photodamage to the organelles would result in a controlled cell death, like apoptosis or paraptosis [15], rather than a mutagenic process in the nuclei. Controlled cell death results in the lack of tissue necrosis, which, despite its assets, risks an uncontrolled inflammation response [16]. Moreover, the dye should be characterized by good photostability to prevent photobleaching [13], which is a process of decomposition and loss of fluorescence and occurs when the dye’s polymethine chains are oxidized by singlet oxygen species [17]. Interestingly, studies by N.S. James [18] showed no quantitative relation between the photobleaching of the PS and effectiveness of the therapy. The phenomena might be explained by the fact that, aside from the photosensitizer itself, the whole tumor microenvironment undergoes irradiation, leading to the generation of ROS from different sources [19].

### 1.4. Cellular Effects of PDT

The localization of the PSs is crucial when considering the efficacy of the PDT. There are agents that target mitochondria, lysosomes, the endoplasmic reticulum, Golgi apparatus, plasma membranes or combinations of these sites and condition various cytotoxic mechanisms. A target location in lysosomes and mitochondria was noticed to be associated with higher PDT efficacy [14]. Interestingly, Kessel et al. demonstrated that autophagy can offer cytoprotection after mitochondrial photodamage. This process can be avoided by targeting the lysosomes, which potentiates apoptosis [14], whereas ER photodamage primarily evokes paraptosis [15,20]. Castano et al. summarized the effects of the photosensitizer: organelle interactions and proved photosensitizers induced cytotoxicity in different ways by targeting the specific cellular compartment [21]. Our study revealed that the cytotoxic and genotoxic effects varied depending on the plasmalemmal or intracellular localization of the PSs [22]. Mainly, irradiation of a culture of melanoma cells right after the administration of curcumin as a drug yielded the highest cytotoxicity, yet with the increase in incubation time toxicity to melanoma cells decreased as the drug moved to the intracellular membranous compartments. The mechanism of melanoma cell death, apoptosis, was proved to be mediated by the caspase-12. Surprisingly, even though cytotoxic tendencies were similar among cancerous and non-cancerous cells, the genotoxicity in the culture of normal human fibroblasts was significantly lower than in melanoma cells. Moreover, the cytotoxic effect of cyanine dyes can differ depending on the resistance of a cell culture to certain chemotherapeutics. Kulbacka et al. showed that the doxorubicin-resistant cell line of human breast adenocarcinoma presented a weaker dye distribution than wild-type cell lines, and after irradiation the therapy was significantly more effective among the resistant cells, suggesting that PDT using such dyes may offer an alternative treatment for multidrug-resistant tumors [23]. 

Characterization of new photosensitizing agents often involves assessing sites of sub-cellular localization. Cyanine dyes were also studied in case of cellular localization-related death induction. First, Delaey et al. showed that, depending on the partition coefficient, different cyanine dye subgroups concentrate more (~1.5) or less intracellularly [24]. The study proved that hydrophobic cyanine dyes, like indocyanines, localize less intracellularly. Murakami et al. proved that bichromophoric cyanine dyes localize preferentially in mitochondria. In the study, high phototoxicity to melanoma was proven to be the result of the interaction between cyanine and mitochondria [25]. Other studies also proved the potency of cyanine dyes in targeting mitochondria [26].

### 1.5. Vascular Effects of PDT

A specific and important trigger for cancer cell death mediated by photodynamic therapy is the anti-vascular effect. It is known that the application of ICG in PDT reduces perfusion by photocoagulating blood vessels [27]. This indirect pathway of cancer eradication additionally enhances the efficacy of the therapy. Shafirstein et al. observed how the vasculature surrounding the tumor tissue was impaired after applying ICG in PDT of a murine mammary carcinoma. Both phototoxic and significant photothermal effects of the therapy resulted in necrosis of the endothelial cells and the deposition of fibrin within the blood vessels [28]. Therefore, an anti-vascular effect is not the result of blood-vessel destruction alone. As the endothelial cells are damaged, they release clotting factors that lead to the formation of thrombi, constriction and occlusion of vessels [29]. In case of large tumors, treatment with PDT might lead to hemorrhaging, and additional side effects of the therapy. These processes deprive tumor cells of necessary nutrients and oxygen. Nonetheless, the resulting hypoxia may also have a negative impact on the therapy. Photosensitizers currently in use require oxygen to create free radicals [30]. Insufficient oxygenation, characteristic of the tumor microenvironment (TME) [31] and intensified by oxygen-dependent PDT [30], directly reduces its efficacy. Moreover, hypoxia stimulates the release of angiogenic growth factors that lead to neovascularization. To inhibit such a process, angiogenesis inhibitors could be combined with PDT to minimize the risk of tumor recurrence [32].

### 1.6. Interstitial PDT

Intratumor light delivery by one or more laser fibers inserted into the target tissue (typically tumor and margins (interstitial PDT, I-PDT)) is applied to activate PSs in deeply seated tumors or tumors more than 10 mm in thickness. If a tumor is too large for the light to be delivered into its entire volume, interstitial PDT (iPDT) could destroy it to the margins of healthy tissue [33]. Moreover, the application of fibers under the guidance of ultrasound or MRI could minimize light scattering by healthy tissue, thus allowing the iPDT to reach the target location and facilitate the eradication of a deep-seated tumor [34] as in pancreatic cancer [35]. According to Chang et al. disulfonated aluminum phthalocyanine (AlS2Pc) application in PDT using fibers placed under ultrasound guidance proved to be effective against prostate cancer by creating lesions up to 12 mm wide. Moreover, the basic connective tissue architecture of the organ and the prostate capsule healed properly, proving that PDT is a less-invasive treatment in comparison to radical surgery [36]. The selective delivery of light could simplify therapies in areas where any excess tissue loss could lead to serious disabilities [37]. An example for such a location is the head and neck, where iPDT using dyes like Foscan [38], or porfimer sodium [37] has already proven to be effective and cause few side effects.

## 2. Cyanine Derived Dyes

**Figure 3:**
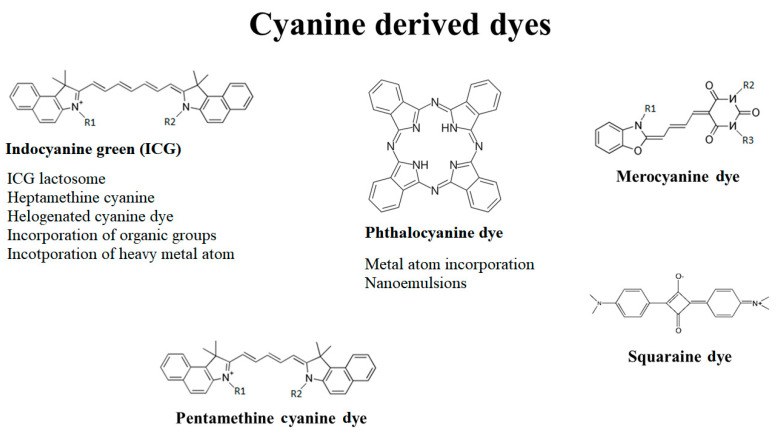
Cyanine-derived dye division and backbone structures. R1, R2 and R3 represent the organic substituent groups.

Cyanine dyes consist of two-terminal heterocyclic units linked by a polymethine bridge core structure (Figure 3) [39]. Over the last few years, the cyanine dyes and their derivatives were widely analyzed for the cytotoxic activity against wide spectrum of tumors. It has been reported they meet most of the requirements for being used in PDT against deep-seated cancers. Each of the main subdivisions of the dyes was described in the following paragraphs. 

### 2.1. Indocyanine Green

One of the most promising cyanine dye is a carbocyanine, widely known as indocyanine green (ICG), the only cyanine dye approved by the Food and Drug Administration (FDA). Due to its absorbance maximum in NIR, prominent fluorescent properties and low dark toxicity, the dye is applied to diagnose liver, cardiovascular and sentinel lymph node pathologies [40,41]. ICG has a high affinity for plasma proteins and is cleared from the body through the biliary pathway, which allows for its use in diagnoses of liver and bile duct functions [42]. Several studies have proven ICG’s toxicity towards various cancer cells. For instance, ICG-mediated PDT induced a 38% decrease in choroidal melanoma viability in six months [43]. Here, it is mostly retained in the Golgi apparatus, endoplasmic reticulum, mitochondria and lysosomes [44]. Additionally, ICG proved to be an efficient photothermal agent, suppressing tumor growth under repeated NIR-light irradiation. The process not only includes the induction of oxidative stress but also the production of heat by converting approximately 88% of the absorbed light into radiation [45]. 

However, the dye also has several disadvantages. These include low photostability, a high level of photobleaching and no specificity towards cancer cells [46]. Of note, the energy yield from the photodynamic reaction is lower than in the PSs characterized by lower absorbance maxima [42,47].

### 2.2. ICG Lactosomes

Studies show that combining high-mass lactate-derived polymers with ICG in ICG–lactosomes (a core-shell-type polymeric micelle or “nanocarrier”) results in greater toxicity towards cancer cells in PDT than by using ICG alone. This tendency was observed in vivo on BALB/c nude mice transfected with human hepatocellular carcinoma (HCC) cell line HuH-7 [48] and gallbladder cancer NOZ cell lines [49]. In the latest case, ICG–lactosomes were effective not only as photosensitizers but also as fluorescent diagnostic agents because of their selective accumulation in cancer tissue, strengthened cytotoxicity and relatively high fluorescence.

### 2.3. Heptamethine Cyanine Dye

Heptamethine cyanine dye, Cy7 is an ICG derivative that has maximum absorbance in the near-infrared range (NIR) and shows high selectivity for cancer cells. On a cellular level, the dye is transported through organic anion-transporting polypeptides (OATPs). The expression of OATPs is regulated by hypoxia-inducible factor 1α (HIF1α), which may possibly explain its selectivity for tumor cells [50]. Moreover, Usama et al. proved, that Cy7 cyanine dyes form covalent albumin adducts that can generate long-lasting intratumor fluorescence due to their enhanced permeability and retention in the tumor tissue [51]. Heptamethine dyes aggregate primarily in the mitochondria and lysosomes. Such localization favors inducing apoptosis over necrosis, thereby minimizing the risk of uncontrolled immunological reaction provoked by necrosis of the affected tissue [16]. 

Cy7 is a promising photosensitizer, diagnostic agent and nanocarrier transporter. Under near-IR light this cyanine derivative might transport anti-cancer drugs exclusively to the tumor [52]. Moreover, Jiang et al. observed that Cy7 conjugated with Gemcitabine displayed relatively high residency time in tumor tissue [53]. Namely, Cy7 remains in a tumor 5–20 days in comparison to ICG, which is removed from the body within 24 h [54].

The disadvantages of heptamethine cyanine derivatives include unfavorable hydrophobicity, which leads to its aggregation in body fluids, extensive photobleaching and insufficiency in ROS production similar to ICG.

### 2.4. Halogenated Cyanine Dyes

Due to the heavy atom effect, the production of free radicals can be significantly increased by halogenation of the cyanine dyes [55]. Atchison et al. established that iodinated IR-783 derivative presents high efficacy towards BxPC-3 and MIA PaCa-2 pancreatic cancer cell lines [35]. Halogenating the dye resulted in significant suppression of tumor growth. Moreover, the viability of MIA PaCa-2 cells after PDT was less than 10% and BxPc-3 was lower than 40%. Interestingly, after irradiation of the same cell lines with ICG, no photodynamic effect was observed. The potential of the derivative was proven in studies where PDT was more effective than a 5-FU treatment of pancreatic cancer. The compounds may also inhibit the growth of cancer. Namely, in a murine model transfected with a human xenograft of BxPC-3 Luc, the untreated pancreatic tumor grew to about 500% of the initial tumor size, whereas the one irradiated with iodinated cyanine IR-783 increased its volume by only about 39% [35]. The study presents PDT as a prospective neoadjuvant and palliative therapy against highly aggressive tumors.

Cao et al. modified the ICG derivative Cy7 with heavy atom iodine to form the novel NIR dye CyI [56]. They investigated the application of PDT simultaneously with photothermal therapy in the study of HepG2 cancer cells [56]. The iodinated dye enhanced the production of ROS production. In this case, the addition of photothermal therapy (PTT) had a synergistic effect on the treatment and strengthened the suppression of tumor growth.

However, the iodinated ICG derivative CyI proved to have poor solubility and tumor-targeting abilities in clinical application [56]. To overcome the problems the dye was modified by PEGylation and the addition of hyaluronic acid, which increased solubility in water [8]. 

Another iodidinated cyanine dye, IR-780, accumulates preferentially in tumor tissue after an intravenous injection [57]. The derivative displays a significant in vivo ability to target tumors. The process is dependent on the cancer’s energetic metabolism, plasma membrane potential and expression of OATPs [58]. Wang et al. showed no significant difference between laser-irradiated and non-irradiated cells treated with IR-780, which is attributed to its inherent toxicity in the dark [59].

By modifying the heptamethine cyanine dye, Noh et al. developed the mitochondria-targeting photodynamic therapeutic agent MitDt-1 [60]. Bromination of the indoline group of the heptamethine dye proved to increase the production of ROS considerably. MitDt-1 accumulates primarily in mitochondria, thus inducing apoptosis. Also, the derivative containing triphenylphosphonium (TPP) and quaternary ammonium enhanced the dye’s solubility and selectivity for the mitochondria of the cancer cells. Moreover, the high toxicity of MitDt-1 toward cancer cells was proven in studies on MCF-7 breast cancer cells in vitro and on NCI-H460 lung cancer both in vitro and in vivo [60].

The analog of IR-780 named IR-808 (MHI-148), was designed, synthesized and screened by Tan et al. [61]. Its photostability and photocytotoxicity was evaluated on the human cervical cancer cell line HeLa and Lewis lung carcinoma (LLC) in mouse xenografts. IR-808 displayed selective aggregation in tumor cells, distinct optical properties and high photostability in serum. Both IR-808 and IR-783 accumulate primarily in mitochondria and lysosomes [62]. Furthermore, IR-808 showed a significant dose-dependent phototoxic effect and distinct suppression of tumor growth after irradiation [62]. In the histopathological examination of the experimental mice, no aggregation in systemic circulation and interstitial fluids were detected. Further investigations into IR-808 proved a high selectivity for cancer and thus potential for imaging gastric [63], prostate [62] and kidney cancer [64].

### 2.5. Incorporation of Organic Groups

Further modifications of IR-808 aimed to increase its water solubility, which included replacing of one of its side chains with (CH_2_)_4_SO_3_ in DZ-1 derivative [65]. To prove the potential of the new derivative, ICG and the newly synthesized fluorophore were compared. ICG itself has high selectivity for HCC with a high tumor to-background ratio (255:1) [66], making it an important tool for identifying HCC lesions during surgery [67]. However, the fluorescence displayed by DZ-1 was significant and lasted longer in contrast to ICG, which allowed for the identification of smaller tumor regions. Moreover, the uptake of the dye in vivo was assessed on HCC Hep3B-Luc cell line xenografts and male New Zealand rabbits. In this case, DZ-1 displayed no accumulation in liver or lung tissue, proving that DZ-1 has a higher specificity for cancer cells than ICG does.

A study by Yang et al., presented the modified heptamethine dye by the addition of 4-amino-2,2,6,6,-tetramethylpiperidine-N-oxyl [68]. The dye was highly effective in ROS production. Moreover, this derivative, first introduced by Jiao et al. [69], has a long triplet-state lifetime and the incorporation of sulfonic acid improves its water-solubility. Additionally, the dye prompted a significant apoptosis of the HepG2 cells after NIR irradiation and showed low dark toxicity.

### 2.6. Incorporation of a Heavy Metal Atom

Incorporation of a heavy metal into the structure of the dye enhances crossing due to the heavy metal effect [70]. Nevertheless, the introduction of the heavy atom in a cyanine dye poses a risk of enhancing dark toxicity [68] and accumulating in healthy tissues [71]. Conjugation of ICG with Au-based nanomaterials sufficiently enhanced the absorption, emission and stability of the fluorophore [72]. It simultaneously induced PTT and enhanced ROS production in PDT, efficiently killing A549 malignant cells [72]. Similarly, the conjugate of ICG with gold–gold sulfide, where gold also acted as an agent for PTT, presented greater stability and improved cytotoxicity towards a HeLa cell line [73]. In addition, silver was incorporated into ICG nanoparticles, resulting in a synergistic interaction between the photothermal effect and PDT. In the study performed by Tan et al., PEGylated silver nanoparticles with a polyaniline shell acted as nanocarriers for ICG and efficiently induced hyperthermia in HeLa cancer cells. In this case, standalone ICG was responsible for fluorescence and phototoxicity [74].

The platinum (II) complex of heptamethine cyanine, IR797-Platin, proved to have extremely high cytotoxicity under NIR-light conditions towards C-33 A (cervical cancer) and MCF-7 breast cancer cell lines [75]. The drug’s cytotoxicity was shown by the photosensitivity of IR797 and the inhibition of DNA transcription and replication by platinum [76].

Zhao et al. synthesized the CYBF2 agent by incorporating: boron difluoride (BF2) into the core structure of cyanine [17]. BF2 reduced the dye’s electron density, resulting in the enhanced photostability. The drug accumulated in mitochondria, induced apoptosis and was efficiently absorbed by MCF-7 cells. Furthermore, the compound presented low dark toxicity and a high level of ROS burst induction. 

### 2.7. pH-Sensitive Cyanine Dyes

Heptamethine cyanine dyes were further modified to be pH sensitive, which eventually increased their tumor specificity [39]. Apart from a relatively high cancer cell selectivity the dye induced fluorescence only after it was placed in an acidic environment. Since extracellular cancer fluid has a high concentration of lactic acid, such a modification made it possible to visualize cancer cells and destroy them more accurately. One of the studied dyes, IR2, incorporated a dimethylamine group that worked as an intramolecular charge transporter. Selectivity was proven by a higher uptake in cancer (HepG2 and HeLa) cell lines than normal cells. Also, the apoptosis following irradiation with the drug was much higher among cancer (80%) than normal (HL-7702) cells (9.4%). Interestingly, cell death was mostly the effect of hyperthermia, not photodynamic reaction.

Siriwibool et al. synthesized a pH switchable dye I2-IR783-Mpip [77] composed of IR783 and N-methylpiperazine. In acidic conditions, the color changes from blue to red, but only the red dye can absorb LED light and displays high toxicity towards HepG2 cells. Cancer cell viability in a neutral environment was about 30% and in an acidic environment decreased to 10%. In this case, death was primarily the result of a free radical production, not the photothermal effect.

Another pH switchable agent was presented by Meng et al. who conjugated 5’-carboxyrhodamines (Rho) and heptamethine cyanine IR765 (Cy) [78]. The newly synthesized conjugate (RhoSSCy) had enhanced fluorescence in a decreased pH value and displayed high stability in pH ranging from 5 to 9. The dye accumulated specifically in tumor cells, presenting a significant fluorescence. In the xenograft studies, the phototoxicity towards cancer was high, considerably increasing the survival rate of mice transfected with MCF-7 cells.

### 2.8. Near-Infrared II Dyes 

Further studies of cyanine dyes were aimed at a red-shifted long-wavelength absorption maximum. Unlike the most dyes currently used in PDT, the PSs that absorb energy into the NIR I range show enhanced tissue penetration. Dyes activated with NIR-II light (1000–1700 nm) were analyzed to lessen the scattering of the light by the tissue, enhance the image contrast and improve the deep-seated tumor detection. The NIR-II dyes, which emit light at a wavelength longer than 1000 nm, were first reported by Antaris et al. [79], followed by the discovery of cyanine dye far-red emissions by Zhu et al. [80]. ICG and IRDye800 were shown to possess emission across NIR-I and NIR-II, marking them as promising and highly specific fluorophores for surgical procedures [42]. The photophysical mechanism of NIR-II emission relies on twisted intramolecular charge transfer (TICT). Further, Starolski et al. observed that the contrast-to-noise ratio (CNR) of the ICG window is twice as high in NIR-II than in NIR-I [81]. Ge et al. studied the efficacy of NIR II-emitting polymer nanoparticles: AuNR vesicles (Ru-complex and a cyanine dye (IR 1061)) [82]. Irradiation by NIR II light induced the release of the Ru complex and generated cytotoxic ^1^O_2_. The therapy efficiently killed the MCF-7 breast cancer cells both in vitro and in vivo.

## 3. Pentamethine Cyanine Dyes

Pentamethine cyanine fluorophores were designed to have tissue-specificity and localize primarily in the adrenal and pituitary glands, pancreas and lymph nodes. Their synthesis aimed to give a promising contrast agent for intraoperative imaging of glands [83]. Newly obtained symmetrical penthametine cyanine dyes, based on a benzoindoleninic ring were tried on a human fibrosarcoma cell line (HT-1080) by Ciubini et al. [84]. The dyes remained active at relatively low concentrations (10 nM), and the drugs were rapidly internalized by cancer cells, which led to high ROS burst. Surprisingly, brominating the benzoindolenine did not produce any increase in free radicals.

2-quinolinium pentamethine carbocyanines were analyzed by Ahoulu et al. on an ES2 ovarian carcinoma cell line [85]. The dye, which was brominated at the mesocarbon remained highly phototoxic and reduced cell line viability from 100 ± 10% to 14 ± 1% after irradiation at a 694 nm wavelength. The compound was characterized by great stability, little dark toxicity and displayed DNA-cleavage. Dye localized mainly in the cytosol and perinuclear regions, whereupon it generated hydroxyl radicals after irradiation.

## 4. Carbocyanines against Drug-Resistant Cancer Cells

PDT may act as a prominent treatment for tumors with multiple drug resistance (MDR). Kulbacka et al. investigated four different cyanine dyes: two carbocyanines: HM-118, KF-570, merocyanine FBF-749 and pyridine-thiazolidine ER-139 combined with PDT against malignant breast adenocarcinoma cell lines. One of the latter was resistant to doxorubicin (MCF/DX), but the other wasn’t (MCF-WT) [86]. Carbocyanines had the greatest phototoxicity. After irradiation with HM-118, 100% of apoptotic cells were detected in both cell lines. When using KF-570, 98% of the wild-type cells and 95% of the doxorubicin-resistant cells remained apoptotic. Other dyes had a much lower apoptotic effect, and their phototoxicity was insufficient. After irradiation with HM-118, overexpression of AIF, a protein involved in caspase-independent cell death, was detected in MCF-7/WT cells [87]. Conversely, in doxorubicin-resistant cells, the protein was absent.

## 5. Squaraine Dyes

Squaraine dyes possess several unique properties, such as significant fluorescence, distinct stability and absorbance wavelength maxima of 600–800 nm. The disadvantages of the dyes involve low solubility and low ROS production following PDT. Fernandes et al. incorporated the sulfur atom into the indolenine-based squaraine core and assessed the potency of the drug in combination with PDT on HepG2 and Caco-2 cell lines [88]. ROS production was enhanced after dithiosquaraine dyes aided PDT. Conversely, monothiosquaraine dyes turned out to be ineffective. Despite high phytotoxicity of dithiosquaraine dyes, the compounds degraded easily and aggregated in aqueous media.

### 5.1. Dicyanomethylene Squaraine Dyes

Martins et al. proved the significant phototoxicity of dicyanomethylene squaraine cyanine dyes against Caco-2 and HepG2 cancer cells in vitro [89]. Despite low singlet oxygen production and moderate light-stability, the chemicals still remained effective. However, further modification of their structure by Wei et al. resulted in dicyanomethylene-substituted benzothiazole squaraines [90]. Among four synthesized dyes, a squaraine derivative with two methyl butyrate sidechains named CSBE showed an excellent phototoxic effect in vitro against seven different cancer cell lines (PC-3, MCF-7, HCT-8, A549, A549T, K562, and LoVo). Negligible dark toxicity was also advantageous. CSBE effectiveness was assessed in xenograft studies, and irradiation following the injection of the dye induced tumor growth suppression. During a histological examination of the liver and kidney, no harm to healthy cells was detected.

Soumya et al. evaluated the potency of symmetrical diiodinated benzothiazolium squaraine (SQDI) dyes in vitro on Ehrlich’s Ascites Carcinoma (EAC) cells [91]. The maximum absorbance of the dye was beyond the NIR of 535 nm. A low concentration of the dye (0.2 mg/mL) induced 100% cytotoxicity after irradiation. Moreover, the dye had no dark toxicity. An in vivo study on Swiss albino mice included the measurement of serum biochemical parameters such as SGPT, SGOT, LDH, CK and ALP after the administration of the dye through the intraperitoneal cavity. None of the parameters increased, meaning that the dye did not extend any toxicity to healthy organs.

### 5.2. Halogenation

Halogenated squaraine dyes were analyzed by Serpe et al. Their efficacy in PDT was tested in vitro on a human fibrosarcoma (HT-1080) tumor cell line [92]. Both brominated and iodinated squaraine dyes proved to induce a significant ROS generation in the first few minutes after irradiation. Despite high initial release of cytochrome c, a drastic reduction was observed 3 h after irradiation. Therefore, in this case necrosis was the main cell death type.

### 5.3. Aminosquaraine Dyes

A study by Lima et al. evaluated the potency of indolenine-based aminosquaraine cyanine dyes as photosensitizers on several cell lines: Caco-2, MCF-7, PC-3. Non-tumor cell lines (NHDF and N27) were controls [93]. Study revealed that, the zwitterionic dye showed high selectivity for the PC-3 cell line in comparison to the normal human cell line. Almost all aminosquaraine dyes, were specifically cytotoxic towards cancer cells and aggregated in mitochondria.

Magalhães et al. have been evaluated the efficacy of several modified zwitterionic dyes on different cancer cell lines (MCF-7, NCI-H460, HeLa, HepG2) and non-tumor porcine liver primary cell culture (PLP2) in vitro [94]. Modifications to the zwitterionic dyes were designed to increase cellular uptake by enhancing their cationic character, increase the red-shift of the dye’s absorption maximum and boost hydrophilicity. All the dyes displayed high phototoxicity towards cancer cell lines, particularly HeLa and MCF-7 cell lines, which showed the highest susceptibility to aminosquaraines. Nevertheless, all dyes showed cytotoxicity against PLP2 cells and a relative inhibition of growth. The use of the aminosquaraine analogues of benzoselenazole was also effective in cancer therapy [95], namely, the inclusion of a heavy metal, selenium, enhanced free radical production while decreasing the dye’s fluorescence emission [96]. The absorbance maximum of the modified dyes was in the 665–685 nm range, and its stability improved significantly. The derivatives were more phototoxic than the benzothiazole analogues, but their toxicity in the absence of light was generally higher as well.

To increase the redshift of unsymmetrical squaraine dyes, Lima et al. incorporated quinoline units into the core structure. Some of the new dyes displayed a wavelength of a maximum absorption at in the far-red spectrum (733 nm) [97]. Despite their limitations (i.e., aggregation in aqueous solution and low ROS synthesis) the new derivatives decreased their dark toxicity and presented a higher cellular uptake because of their cationic character. Although production of singlet oxygen was relatively weak, the dyes showed substantial phototherapeutic activity against breast cancer cell lines (MCF-7 and BT-474). These results were comparable to previously studied indolenine-based aminosquaraine dyes. Further modifications to them that would strengthen their singlet oxygen generation and may lead to their successful application in PDT.

## 6. Merocyanines

Merocyanines raised hope for low-invasive lymphoma [98], leukemia [99] and neuroblastoma [100] treatments as they exhibited high specificity for cancer cells. The compounds are currently undergoing preclinical studies as a treatment for leukemia [99]. The exceptional permeability of the MC540 dye to leukemic leukocytes and immature hemopoietic precursors led to its extensive analysis.

The drawbacks of the group involve maximum absorbance of light outside of the NIR spectrum (556 nm) and preferential peroxidation of phospholipids in the membrane [101]. The latter leads to the induction of necrosis and constraints on the utility of the drugs in vivo. Additionally, their use in PDT against melanoma on the Cloudman S91 cell line turned out to be less effective than the currently applied porphyrin dyes [102]. Nonetheless, the research on merocyanines as a potential hematological cancer treatment hasn’t stopped. A rhodamine complex of merocyanine underwent an in vitro study on K562 leukemia cells and revealed a decrease of cancer cell viability [103]. Apart from cancer treatment, merocyanines are also evaluated as an antimicrobial therapy on *Staphylococcus aureus* [104].

### Immunoregulatory Agent

It has been shown that merocyanines exhibit immunoregulatory properties. The compound group can regulate an immune response by inhibiting T-lymphocyte proliferation and B-cell differentiation. Also, T-cell helper activity can be stimulated [105]. Therefore, merocyanines reveal the potency against leukemia and lymphoma. The drugs may also may find application in graft-versus-host disease prophylaxis and treatment of several autoimmune diseases. Traul et al. evaluated the application of PDT with MHC540 in reducing GVHD in murine models of allogeneic hematopoietic stem cell transplantation [106]. Prior studies reported, that the sensitivity of cells to merocyanines is determined by the dye’s binding to the targeted cells [107]. The binding affinity is reduced in mature lymphocytes and elevated in hematopoietic stem cells. Irradiated lymphocytes displayed no proliferative response after treatment with ConA (concanavalin A), LPS (lipopolysaccharide), PHA (phytohemagglutinin) and IL-2 (interleukin-2). Also the survival of MHC540 treated cells increased by 50–80% [106].

## 7. Phthalocyanines

Phthalocyanines are successful photosensitizers in the therapy of skin malignancies like basal cell carcinoma and diseases like psoriasis [108]. The compounds belong to the group of second-generation photosensitizers present some similarities to porphyrins. In contrast to the latter, they have absorption maxima in the range of 670–780 nm [109].

### 7.1. Incorporation of a Metal Atom

The substitution of the phthalocyanine central atom of with zinc (II), aluminum (III), gallium (III) or silicon results in increased cytotoxicity in biological studies of the drugs [110]. The advantages include high stability, fluorescence [111] and low dark toxicity [112]. Their weakness, however, is hydrophobicity, which leads to aggregation in aqueous media [109]. Zinc (II) phthalocyanine Pc13 induced apoptosis and necrosis triggered by free radical production in B16Fo melanoma cells [113]. The level of apoptosis regulators (Bcl-2, Bcl-xL and Bid) increased after irradiation with the drugs. In addition, permeability of the mitochondria towards the inner-derived ROS was detected. The necrotic pathway resulted from an increase in lactate dehydrogenase concentration in extracellular compartments [113].

To enhance water solubility of the zinc phthalocyanine, sulfonic [114], phosphoric [115] and carboxylic group [116] substituents were introduced. The exposure of cervical cancer cells (HeLa) to sulphated zinc (II) phthalocyanines and irradiation with 673 nm diode laser resulted in DNA fragmentation, membrane damage and effective cytotoxicity. This led to a 25% decrease in cell viability [117]. Aniogo et al. combined sulfonated zinc (II) phthalocyanine with doxorubicin and observed a synergistic cytotoxic effect on MCF-7 cell lines [118]. To improve cytotoxicity and penetration of phthalocyanine derivatives into cancer cells, the cyanines might be conjugated with chemotherapeutical drugs. For instance, Al (III) phthalocyanine chloride tetrasulfonic acid (AlPcS4) with different chemotherapeutic agents was studied on gastric cancer cells [119], and zinc phthalocyanine with doxorubicin acted against SK-MEL-3 melanoma cells [120].

### 7.2. Nanoemulsions

Because phthalocyanines are lipophilic, L.A. Muehlmann developed nanoemulsions that can transport aluminum-phthalocyanine chloride (AIPc) to cancer tissue. The approach prevented aggregation in aqueous media [121]. The nanoemulsions are mostly composed of castor oil, Cremophor ELP^®^ and a monodisperse population of nanodroplets. Cell viability of mammary MCF-7 adenocarcinoma cells significantly decreased and the production of LDH excessively increased. Conversely, without such emulsions AIPc was ineffective. The complex dye aggregated mostly in the cytoplasm and outside the nucleus, thus inducing no damage to the DNA and preventing genome modifications. Subsequently, nanoemulsions of aluminum-phthalocyanine were studied in vivo in a PDT against 4T1 breast adenocarcinoma tumor [122]. The primary breast tumors were eradicated after application of PDT, and contrary to the untreated mice group, metastases to the lungs were not observed.

## 8. Conclusions

The absorbance maxima of cyanine dyes lie within the NIR-I spectrum and the light used to activate them penetrates deeper into the tissue. They exhibit significant fluorescent properties and thus are applied in photodynamic diagnostics. ICG is an FDA approved dye which has for decades been clinically used as a diagnostic agent. Heptamethine dye is exceptional for cancer cells selectivity. It is transported through organic anion-transporting polypeptides (OATPs) the production of which increases in cancer cells. Certain cyanine dyes (ICG and IRDye800) can emit light at an NIR-II wavelength of 1000–1700 nm. Such a characteristic could provide a better contrast-to-noise ratio (CNR) diagnostic image and higher specificity for tumors [81]. 

Cyanine dyes do have their weaknesses: poor water solubility and low ROS generation. Nonetheless, the ROS production can be increased by incorporating certain organic groups, heavy atoms, halogenation or metal atoms [55,56,60,72,73,92,96]. This review of the use of cyanines and their derivatives as potential photosensitizers indicates that they could be efficiently activated by light, causing the death of target cells [35,48,49,56,62,68,82,85,89,122]. Cyanine dyes provoke cell death primarily through apoptosis [2,17,69], which benefits the therapy because it prevents an excessive inflammatory response. To overcome the problem of poor water solubility the dyes can be further modified by, for example, incorporating organic [8,69] or other groups [114,115,116]. Specificity for cancer tissue can be improved by sensitizing cyanine dyes to pH and enhancing their phototoxicity in an acidic environment, which is characteristic for extracellular cancer fluid [39,78].

Moreover, merocyanines, are especially worth mentioning for their unique immunoregulatory properties, namely, the ability to interact with lymphocytes [105]. Currently, merocyanines are in preclinical studies focused on treating leukemia [99]. 

Current studies involving numerous cyanine dyes show that they may enrich the stock of the permanently used pool of agents in PDT. Considering the very high cost of introducing new agents, it is important to show persuasive evidence of their new and remarkable properties. Further studies to exploit the advantages of cyanine dyes for therapeutic possibilities in low-invasive cancer treatments include absorbance within the NIR, low dark toxicity and fluorescent properties, combined with the application of optical fibers.
